# A survey on surgeons' perceived quality of the informed consent process in a Swiss paediatric surgery unit

**DOI:** 10.1186/s13037-015-0076-3

**Published:** 2015-08-28

**Authors:** Julie Guinand, Christophe Gapany, Jeanne-Pascale Simon, Jean-Blaise Wasserfallen, Jean-Marc Joseph

**Affiliations:** Peadiatric surgery Department, Centre Hospitalier Universitaire Vaudois, Rue du Bugnon 46, 1011 Lausanne, Switzerland; Peadiatric Surgery Private Practice, 1003 Lausanne, Switzerland; General Management of the University Hospital, Centre Hospitalier Universitaire Vaudois, 1011 Lausanne, Switzerland

## Abstract

**Aim:**

To evaluate the levels of satisfaction and opinions on the usefulness of the informed consent form currently in use in our Paediatric Surgery Department.

**Materials and methods:**

Design: Qualitative study carried out via interviews of senior paediatric surgeons, based on a questionnaire built up from reference criteria in the literature and public health law.

**Results:**

Physicians with between 2 and 35 years experience of paediatric surgery, with a participation rate of 92 %, agreed on the definition of an informed consent form, were satisfied with the form in use and did not wish to modify its structure. The study revealed that signing the form was viewed as mandatory, but meant different things to different participants, who diverged over whom that signature protected. Finally, all respondents were in agreement over what information was necessary for parents of children requiring surgery.

**Conclusion:**

Paediatric surgeons seemed to be satisfied with the informed consent form in use. Most of them did not identify that the first aim of the informed consent form is to give the patient adequate information to allow him to base his consent, which is a legal obligation, the protection of physicians by the formalisation and proof of the informed consent being secondary. Few surgeons brought up the fact that the foremost stakeholder in paediatric surgery are the children themselves and that their opinions are not always sought. In the future, moving from informed consent process to shared decision-making, a more active bidirectional exchange may be strongly considered. Involving children in such vital decisions should become the norm while keeping in mind their level of maturity.

## Introduction

During the 20th century, the issue of patient involvement in the treatment decision process has gradually become a necessity in adult [1] and paediatric patient [2]. In Switzerland, the concept of informed consent first appeared at the end of the 1970s, with patient information being a central element [3]. No legislation regulating the relationships between healthcare professionals and their patients existed in Switzerland before 1982 [4]. Judges had to make up rules to resolve disputes as equitably as possible. This led to a tendency to obtain formal, written informed consent from patients, especially in hospitals [5, 6]. Previously, agreement between doctors and patients was mainly verbal, and the parties often considered consent to be implicit from the moment the patient presented himself for consultation or intervention.

The University Hospital of Lausanne (CHUV) followed this trend, and the topic of patient information is regularly discussed. Over the years, disease-specific patient information leaflets have been drawn up, and informed consent forms are in use in all departments. However, the practical application of informed consent forms may not be optimal yet.

A number of studies have dealt with the contents and different types of preoperative information given to patients, but few have dealt with the qualitative side of informed consent forms from the physician’s perspective. The aims of this study were to evaluate surgeons’ levels of satisfaction with their own department’s informed consent form, and to evaluate its usefulness and practical value.

## Material and methods

### Population

All, senior paediatric surgeons and senior registrars of the CHUV Paediatric Surgery Department and of the Children’s Hospital of Lausanne were included in the study.

### Study design

Participation was proposed via an email explaining the study’s aims regarding preoperative written informed consent. Respondents were interviewed using a questionnaire and their answers were transcribed for comparison. Respondents did not receive the questionnaire in advance and were not given time to prepare their answers. The goal was to elicit the participants’ spontaneous answers whilst guaranteeing anonymity and confidentiality. The questionnaire was put together using reference criteria from the literature and the Cantonal Law on Public Health of Canton of Vaud [5, 6, 7] which states that patients must at least have an understanding of f the goals and risks of the operation.

### Measurement methods

The questionnaire was made up of three sections including open, semi-open and closed questions:

- Section one asked paediatric surgeons for their opinions on the informed consent form currently used by the CHUV’s Paediatric Surgery Department.
- Section two assessed the adequacy of the current means used to obtain parental consent and focussed on the administrative processes surrounding the informed consent form.
- Section three asked for an appreciation of the information which surgeons felt necessary to provide to parents concerning their child’s operation. In this section we investigated if the surgeon’s information was simple, understandable, appropriate and honest as demanded by the Cantonal on Public Health (Vaud) and a Federal Court Judgement (105 II 284).

## Results

Out of twelve paediatric surgeons working in the Department, eleven responded, resulting in a participation rate of 92 %. Two respondents were interviewed by telephone. Respondents had between 2 and 35 years experience in this field (median 8 years).

### Result from survey on the informed consent form

Survey answers on the informed consent form are given in Table 1. Ten out of eleven surgeons agreed on its definition and considered that the form was indeed proof that information and explanations had been given to parents about their child’s operation. Only two interviewees mentioned any doubt about the form’s legal value. Eight out of eleven surgeons seemed to appreciate the form’s layout and structure. It was viewed as straightforward, and seven surgeons (63 %) had no wish to change that structure. Suggested modifications were minor:

**Table 1:** Result of survey on the informed consent form used

Opinion	>75 %	>50 %	>25 %	<25 %
Definitions: what is the informed consent form?	Defined the informed consent form as proof that information and explanations were given to parents	-	-	-
Questionnaire structure	-	Satisfied with the form they use	Mentioned the mandatory character of the form	A legal requirement; induced by the consent form; biased relationship
Subjective opinion on parents’ understanding of the form	Think that the form is easy to understand	-	-	-
Subjective opinion on parent’s understanding of the form’s usefulness	Parents understand the form’s usefulness when it is explained to them	-	Think that the form is poorly understood	-
Potential improvements	-	Would not make any changes	-	Get rid of the form; adding a note for the non-signing parent; Use the institutional form as a template

- adding a note for the non-signing parent should they be divorced or separated was advocated by one surgeon
- using the institutional form mentioning only the description of the operation, that information was given and the name of the surgeon

Despite of this favourable assessment, more than 25 % of surgeons thought that parents failed to grasp the form’s usefulness and that this could only really be improved by verbal explanations about the operation itself.

### Administrative processes

All respondents mentioned the fact that signing the informed consent form was mandatory, but they did not agree on the reasons. All respondents give the information at the preoperative consultation and nine out of 11 (81 %) are present at the moment of the signature. Eight out of eleven (72 %) never forget to have the form signed, and three occasionally forget (28 %). The most frequent answer was that it was an institutional requirement (table 2). This type of answer was most frequent noticed in surgeons who had fewer than 10 years surgical experience. All participants were aware of the existence of an institutional guideline, but only one claimed to know its content.

**Table 2:** Administrative process to obtain inform consent from the relative by the paediatric surgeon

Administrative processes	>75 %	>50 %	>25 %	<25 %
When is the form signed?	At the preoperative consultation (for elective surgery)	-	-	Mentioned potential emergency situations
Are surgeons present at signature?	Yes	-	Occasionally	-
Is a signature ever forgotten?	-	Never	Occasionally	-
Is signing an informed consent form mandatory?	Yes	-	-	-
Do surgeons know why informed consent forms must be signed?	-	Entering the operating room without written informed consent is not allowed	-	Institutional requirement

**Fig. 1:**
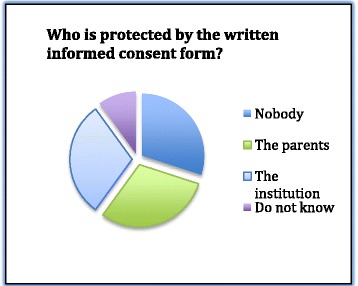
Knowledge of protection value of the inform consent form by surgeons

The widest variety of answers came when surgeons were asked whom the written informed consent form protected. This question splits them into four groups: One did not know, three declare that the form does not protect anybody, three the parents and four the institution (Fig. 1).

### Applying the informed consent form in practice

**Fig. 2:**
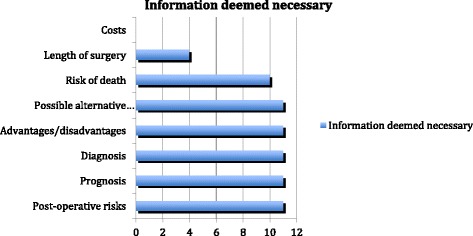
Information deemed to be given by surgeons before operation

The necessity to inform about diagnosis, prognosis, advantages and disadvantages, possible alternative treatments, and intra- and postoperative risks were mentioned by all respondents. More than 25 % of respondents stated that a list of every potential risk should not be given because it may cause unnecessary anxiety to patients/parents and bias overall fact retention. Ten out of eleven (90 %) raised the necessity of mentioning risk of death even if it is only brought up in cases of extremely risky operations. The length of surgery was mentioned by four surgeons (36 %). None of them mentioned the cost of the operation (table below). Economic aspects were never brought up as even the surgeon did not know the final cost of a given operation (Fig. 2).

Eight out of eleven users (72 %) appreciated the empty space left for additional information.

The two most frequent surgical complications- haemorrhage and infection- are already printed on the form. Surgeons unanimously responded that this did not reduce the probability of other risks being discussed. However, it did allow them to bring up these prominent risks without having to dwell on them.

## Discussion

Parents’ signature on an informed consent form is a rule which is likely to become mandatory in most medical institutions in the coming years. From a legal point of view, the physicians carry the burden of proving that a patient received all pertinent information and has agreed on the proposed procedure. The specific informed consent form evaluated in this study has been in use since July 2010. As it became mandatory in March 2011, we decided to find out exactly how surgeons used it, whether or not they were satisfied with it and whether the document met all legal and ethical requirements.

**Fig. 3:**
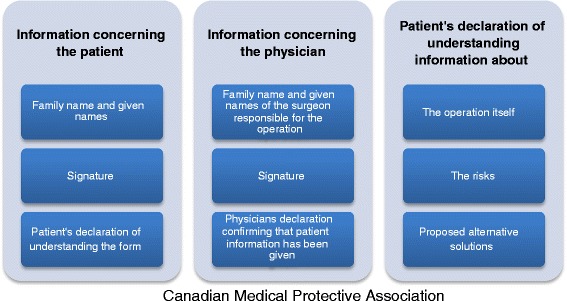
Recommendation of the Canadian Medical Protective Association for a suitable informed consent form

In our paediatric surgery department, since July 2010, written informed consent has been mandatory to move a patient into an operating room. Many patients and parents have limited knowledge about the implications of informed consent forms and fail to realise that they protect their interests first and foremost to be able to exercise their autonomy [8]. The forms constitute a clear, written confirmation that the necessary explanations have been given and that the patient has accepted the treatment [9, 10, 11]. The patient’s signature (or that of their legal guardian) on an informed consent form is also important in order to be able to formalise and thus to prove the occurred agreement to the treatment. A suitable informed consent form should comprise the characteristics summarized in Fig. 3:

According to the Cantonal Law on Public Health (Art. 21) [7] and to Federal Court Judgements (105 II 284), [12] the physician must give the patient simple, understandable, appropriate and honest information concerning the health status:

- the diagnosis
- the prognosis
- the course of medical examination and treatment and their alternatives (type, duration, consequences, advantages, disadvantages and risks)
- the therapeutic procedure
- the economic aspects of the treatment.

The CHUV Paediatric Surgery Department’s current informed consent form has been in use for about 10 years, and has become an internal requirement in July 2010. It is structured to leave plenty of room for supplementary guidance or sketches, in addition to the information on the surgical act itself, potential modifications and complications and the name of the surgeon who informed the patient and/or the legal guardians. The form also has a space for a translator’s signature, if present during the consultation. This feature is specific to our Department and is not included in the standard institutional form.

We found that surgeons did indeed comply with institutional requirements, although not truly knowing what their proper conduct should be. The form was considered to be very informative but participants also identified a risk of depersonalising the doctor-patient relationship.

Except for the economic aspects, paediatric surgeons give their patients’ parents all the information required by law regarding the intervention which they propose. They must respect the Law on Public Health’s Article 21 regarding the right to information. In the end, surgeons tell patients what they know and what they want the patients to know. Mentioning every possible risk would be both impossible and counterproductive: the list would hardly be comprehensive and raise unnecessary concerns for patients and/or guardians [3, 11]. Swiss Public Health laws plan not to pointlessly worry patient.

According to a decision of the Swiss Federal Court (ATF 4C.366, 2006.), risks below 1 % need not be discussed [12]. This decision was based on recommendation of the Swiss Society of orthopaedics and is not an absolute principle.

Nevertheless, in the future, this could become a non-negligible medico-legal issue. As physicians are increasingly challenged by burden of proof issues, it is now almost more common for them to face legal action over failures in their duty to inform patients rather than over medical error.

The informed consent form is not aimed directly at children. It is not structured for their levels of understanding, nor does it require the child’s signature. Even though the concept of maturity or mental age should take preference over biological age, this notion seems to have been forgotten in the form [13]. Surprisingly this was not raised by any of the respondents.

We realise that the results presented here come from a small sample; any interpretation must, therefore, be done with care and without generalisation. The research carried out resembled a qualitative study based on a questionnaire of mostly open questions. Surgeons answered subjectively about a form that they almost know by heart. This context may not be ideal to take a step back and analyse the informed consent form properly.

Shared decision-making (SDM) is a bidirectional exchange of information, taking in account all values and preferences of the patient [14]. It is a more active process, both for surgeon and patient. A recent meta-analysis of 115 trials emphasizes the benefit of SDM with a more accurate perception of risks and benefits, greater comfort with the decision, and higher participation in decision [15]. SDM, designed to become the future gold standard for informed consent appears particularly accurate for paediatric surgery and may strongly be considered in a near future.

It is easy to forget that the child, not the parents, is the person of interest. We believe that the informed consent form does not take enough account of the children and their rights, and we do not know how much information surgeons impart to them. The fact that parents have a significant decisional role increases not only their responsibility, but also their feelings of guilt, should something go wrong. This interesting topic could be the subject of a future study.

## Conclusion

Most of the paediatric surgeons involved in the study did not identify that the first aim of the informed consent form is to give the patient adequate information to allow him to base his consent, which is a legal obligation. The protection of physicians by the formalisation and proof of the informed consent remains being secondary. Few surgeons brought up the fact that the foremost stakeholder in paediatric surgery are the children themselves and that their opinions are not always sought. In the future, moving from informed.

consent process to shared decision-making, a more active bidirectional exchange may be strongly considered. Involving children in such vital decisions should become the norm while keeping in mind their level of maturity.
